# Genomic prediction in insects: a case study on wing morphology traits in the jewel wasp *Nasonia vitripennis*

**DOI:** 10.1093/g3journal/jkaf285

**Published:** 2025-11-29

**Authors:** Shuwen Xia, Gabriella Bukovinszkine Kiss, Hendrik-Jan Megens, Martien A M Groenen, Bas J Zwaan, Piter Bijma, Bart A Pannebakker

**Affiliations:** Institute of Animal Science, Jiangsu Academy of Agricultural Sciences, Nanjing 210014, China; Laboratory of Genetics, Wageningen University & Research, Droevendaalsesteeg 1, Wageningen 6708 PB, The Netherlands; Animal Breeding and Genomics, Wageningen University & Research, PO Box 338, Wageningen 6700 AH, The Netherlands; Laboratory of Genetics, Wageningen University & Research, Droevendaalsesteeg 1, Wageningen 6708 PB, The Netherlands; Animal Breeding and Genomics, Wageningen University & Research, PO Box 338, Wageningen 6700 AH, The Netherlands; Animal Breeding and Genomics, Wageningen University & Research, PO Box 338, Wageningen 6700 AH, The Netherlands; Laboratory of Genetics, Wageningen University & Research, Droevendaalsesteeg 1, Wageningen 6708 PB, The Netherlands; Animal Breeding and Genomics, Wageningen University & Research, PO Box 338, Wageningen 6700 AH, The Netherlands; Laboratory of Genetics, Wageningen University & Research, Droevendaalsesteeg 1, Wageningen 6708 PB, The Netherlands

**Keywords:** genomic prediction, genomic estimated breeding value, accuracy, morphology, wing, tibia, biological control, insect

## Abstract

Biological control is a sustainable strategy to combat agricultural pests. Yet, legislation increasingly restricts importing nonnative biocontrol agents. Thus, selective breeding of biocontrol traits is suggested to enhance performance of existing biocontrol agents. Genomic prediction, where genomic data are used to estimate the genetic merit of an individual for specific traits, is an alternative to exploit genetic variation for the improvement of native biocontrol agents. This study aims to establish a proof of principle for genomic prediction in insect biocontrol agents, using wing morphology traits in the model parasitoid *Nasonia vitripennis* Walker (Pteromalidae). We performed genomic prediction using a genomic best linear unbiased prediction (GBLUP) model, using 1,230 individuals with 8,639 SNPs generated by genotyping-by-sequencing (GBS). We used individuals from 2 generations from the outbred HVRx population, 717 individuals from generation 169 (G169) and 513 individuals from generation 172 (G172). To assess genomic prediction accuracy, we used across generation validation (forward validation for G172 from G169 and backward validation for G169 from G172) and also 5-fold cross-validation. For size-related traits, including tibia length, wing length, wing width, and second moment area, the accuracy of genomic prediction was close to 0 in both across generation validations but much higher in 5-fold cross-validation (ranging from 0.54 to 0.68). For the shape-related trait wing aspect ratio, a high accuracy was found for all 3 validation strategies, with 0.47 for across generation forward validation (AGFV), 0.65 for across generation backward validation (AGBV), and 0.54 for 5-fold cross-validation. Overall, genomic selection in insect biocontrol agents with a relative small effective population size seems promising. However, factors such as the biology of insects, phenotyping techniques, and large-scale genotyping costs still challenge the application of genomic selection to biocontrol agents.

## Graphical Abstract

**Graphical Abstract:**
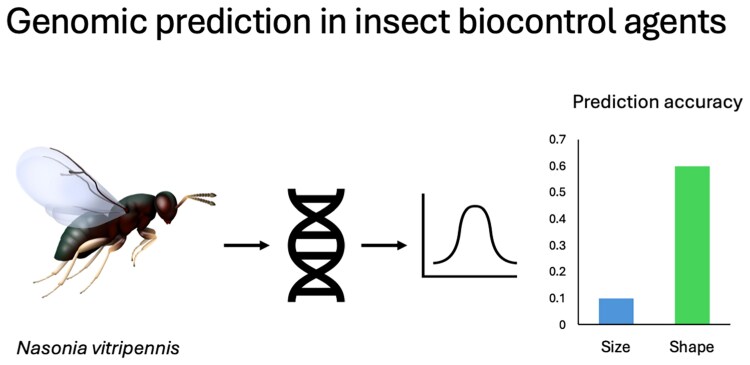

## Introduction

Biological control is a strategy to combat agricultural pests using natural enemies of pest species, such as predators and parasitoid wasps, as biocontrol agents (DeBach 1958; Van Den Bosch 1971). Over the past decades, effective biocontrol strategies have been established, using both native and nonnative natural enemy species (van Lenteren et al. 2011). However, the Nagoya Protocol on Access and Benefit-sharing (https://www.cbd.int/abs) has considerably limited the import of nonnative biocontrol agents (Cock et al. 2010; van Lenteren et al. 2011; Mason et al. 2018, 2023). Thus, optimizing the use of existing and native biocontrol agents became necessary. To enhance the efficacy of existing biocontrol agents, the use of genetic tools has been discussed over the last 50 years (DeBach 1958; Hoy 1986; Bielza et al. 2020; Leung et al. 2020). However, the potential for genetic improvement of biocontrol populations has largely been unexplored (Wajnberg 2004; Lommen et al. 2017). Selective breeding, which uses the presence of standing genetic variation to select for traits of interest in biocontrol populations, may offer an opportunity to improve the performance of natural enemies used in biocontrol.

Genomic selection is a promising selective breeding approach that uses information from genome-wide DNA markers to efficiently select for complex traits (Meuwissen et al. 2001). The availability of large numbers of single nucleotide polymorphisms (SNPs) enabled the use of genomic selection, and it has been extensively studied in livestock and plant breeding over the last decade (Meuwissen et al. 2013). In genomic selection, the quantitative trait loci (QTL) affecting the traits of interest are assumed to be in linkage disequilibrium (LD) with 1 or more SNP markers. The predictor of an individual's genetic merit is the genomic estimated breeding value (GEBV), obtained as the sum of all SNP marker effects of the individual, a statistical method called genomic prediction. GEBVs are then used to rank selection candidates based on their genotype to select parents for the next generation of breeding without phenotypic data. Genomic selection has revolutionized animal and plant breeding, and genomic prediction is increasingly used in human genetics to predict the individual risk of complex diseases (Schaeffer 2006; Hayes et al. 2009; Heffner et al. 2009; Jannink et al. 2010; Wray et al. 2019). So far, however, the potential of genomic selection for genetic improvement of insects has only been sparsely reported (Bernstein et al. 2023; Sandrock et al. 2025).

As a proof of principle of genomic prediction in biocontrol agents, here we focus on its application in a parasitoid wasp. Parasitoids are among the most widely utilized insect biocontrol agents in practice (Waage and Hassell 1982; Strand and Obrycki 1996). The *Nasonia* genus, gregarious parasitoid wasps of blowfly pupae, has been used for over half a century as a model species in developmental and evolutionary genetics (Whiting 1967; King 1993; Rivero and West 2005, 2002; Werren and Loehlin 2009; Werren et al. 2010; Pannebakker et al. 2011). As with all Hymenoptera, *Nasonia* is haplodiploid, with diploid females developing from fertilized eggs and haploid males from unfertilized eggs. It includes 4 species: *Nasonia vitripennis*, *N. longicornis*, *N. giraulti*, and *N. oneida* (Werren et al. 2010). *Nasonia* species are used as biocontrol agents of stable flies (Floate et al. 1999; Kaufman et al. 2001) and as a general model for biocontrol study (Leung et al. 2019; Leung 2024). The best-studied species in the genus is *N. vitripennis*, which has its genome sequence released and annotated. The *Nasonia* genome consists of 5 chromosomes with a total size of ∼335 Mb and ∼446 cM (Werren et al. 2010; Lynch 2015). The availability of a genome assembly has driven the development of a range of genomic and genetic tools, which have facilitated the investigation of gene expression and regulation, and the identification of genes involved in complex genetic traits (Werren et al. 2010, 2016; Pannebakker et al. 2013; Wang et al. 2013, 2015; Ferree et al. 2015; Lynch 2015; Dalla Benetta et al. 2019; Buellesbach et al. 2022). The availability of a genome assembly and other genetic tools makes *N. vitripennis* an interesting model organism to investigate the prospects of genomic prediction in biocontrol agents.

The main goal of this study was to seek a proof of principle for the use of genomic prediction in biocontrol agents, using the parasitoid wasp *N. vitripennis* as a model organism. Importantly, *N. vitripennis* is a gregarious parasitoid, which can lay up to 60 eggs in a single dipteran pupa (Whiting 1967). This can create environmental similarity between offspring developing within the same pupa (here referred to as “host”), which needs to be accounted for in the statistical model to avoid confounding environmental effects with genetic effects. Moreover, it was previously found that much of the phenotypic variance of wing morphology and tibia length can be attributed to the host in which the wasp had developed (Xia et al. 2020). Thus, a common environmental effect (i.e. host effect) should be included in the genomic prediction analysis. To ensure the statistical power of genomic prediction, a sufficiently dense and well-distributed set of markers across the whole genome is required. In many animal and plant species, flexible low-cost high-throughput genotyping arrays have been developed and commercialized for this purpose. However, because of the limited uptake of genetic investigation of biocontrol agents, a high-throughput genotyping SNP array is not available for *N. vitripennis*. As an alternative, genotyping-by-sequencing (GBS) is a genotyping approach to obtain genome-wide marker genotypes from sequence data and has been applied in a variety of breeding schemes, especially in plants (Davey et al. 2011; Elshire et al. 2011; Poland et al. 2012; Poland and Rife 2012; Crossa et al. 2013; De Donato et al. 2013; Wu et al. 2015). Here, we investigate the prospects of genomic prediction based on GBS in insect natural enemies, using tibia length and wing morphology as model traits in *N. vitripennis*. To measure the quality of the resulting GEBVs, we performed different validation strategies, both within and across generations. We found that the accuracy of genomic prediction differed between traits and validation strategies, which could be largely explained by the biology of the species, such as the large effect of the shared host environment, and the study populations used.

## Materials and methods

### *N. vitripennis* stock culture

As our source population, we used the HVRx outbred laboratory population, established from strains collected from a single population in the Netherlands (van de Zande et al. 2013). To preserve genetic diversity across generations, the HVRx stock is maintained according to a fixed schedule, in which approximately 120 mated females were transferred to 4 new mass culture vials to initiate the next generation (van de Zande et al. 2013). Per vial, 50 blowfly pupae (*Calliphora vomitoria* Linnaeus Calliphoridae) were provided as hosts for oviposition. To ensure optimal mixing of the wasps needed to maintain an outbred laboratory population, the parasitized hosts were redistributed over 4 new mass culture vials each generation before offspring emerged. Approximately 14 d were needed to complete a cycle at 25 °C under 16-h light:8-h dark conditions.

### Morphological trait measurements

Because of haplodiploidy, and of large sex-specific differences in wing morphology (males have vestigial wings), only diploid females were used in our analyses. In total, 1,248 females were collected from parasitized hosts randomly isolated from the mass-reared HVRx population across 2 generations: 720 individuals from generation 169 (G169) and 528 individuals from generation 172 (G172). We recorded the host identity for individuals from G172 but not for individuals from G169. The right forewing and right hind tibia were detached and mounted in the mounting medium Euparal (Waldeck GmbH & Co. KG, Division Chroma, Münster, Germany) under coverslips on microscope slides. Slides were photographed on a Zeiss Imager.A1 microscope (Carl Zeiss AG, Göttingen, Germany) at 2.5× magnification. Data for wing morphology were obtained by positioning landmarks on each digitized wing, using tpsDig software (Rohlf 2013), calibrated using a Carl Zeiss Stage Micrometer (5 mm + 100/100, Carl Zeiss AG, Göttingen, Germany), following a previous study (Xia et al. 2020). Coordinates of the landmarks were used to calculate the following wing traits: wing length, wing width, second moment area, and wing aspect ratio (full description of the landmarks in Xia et al. 2020). Descriptive statistics of these traits are given in Table 1.

**Table 1.** Descriptive statistics for tibia length and wing morphology traits measured in *N. vitripennis*.

Traits (units)	Mean			SD			CV (%)			Min			Max		
	G169	G172	Combined	G169	G172	Combined	G169	G172	Combined	G169	G172	Combined	G169	G172	Combined
Tibia length (µm)	603	618	610	42.17	30.09	38.31	6.99	4.86	6.28	401	461	401	692	680	692
Wing length (µm)	1921	2009	1958	118.52	88.67	115.42	6.17	4.41	5.89	1392	1516	1392	2180	2181	2181
Wing width (µm)	874	898	884	57.65	43.98	53.64	6.59	4.90	6.07	644	658	644	999	981	999
Second moment area (mm^4^)	0.43	0.51	0.46	0.10	0.09	0.10	23.42	16.98	21.92	0.12	0.16	0.12	0.70	0.86	0.86
Aspect ratio (-)	2.20	2.24	2.21	0.04	0.03	0.04	1.60	1.56	1.80	2.09	2.12	2.09	2.36	2.35	2.36

For each trait, the table provides the units of measurement, the mean, the SD, the coefficient of phenotypic variation (CV = 100% × SD/mean), the minimum value (Min), and the maximum value (Max).

### DNA isolation and sequencing

After detaching the wing and tibia, the remaining body of each individual female was immediately put into an Eppendorf tube with DNA buffer provided by QIAamp DNeasy 96 Blood & Tissue Kit (Qiagen, Venlo, The Netherlands) to prevent DNA degradation. DNA was extracted by using QIAamp DNeasy 96 Blood & Tissue Kit, following the manufacturer's instructions. Afterward, DNA yield and quality were checked by full-spectrum spectrophotometer NanoDrop 2000 (Thermo Scientific, Waltham, Massachusetts, United States) and Qubit 2.0 fluorometer (Invitrogen, Carlsbad, California, United States). After qualification and quantification, DNA samples were used for GBS to identify SNPs across the genome. GBS is a reduced representation approach that uses restriction enzymes to fragment the genome (Elshire et al. 2011), followed by size selection and sequencing. Here, each individual DNA sample was digested by *ApeKI* restriction enzyme and adapters were ligated to both ends of the DNA fragments (one end containing a barcode to identify the individual sample, the other without). After samples were pooled and cleaned, fragments with different adapters at both ends and with a size between 170 and 350 bp were amplified by polymerase chain reaction (PCR); and sequenced on the Illumina HiSeq X Ten platform (150 bp paired-end, Illumina, San Diego, California, United States). Sequence reads are deposited in the NCBI Short Read Archive under BioProject accession number PRJNA1188512.

### Alignment, variant calling, and filtering

After reads were sorted by individual and DNA barcodes were removed, cleaned sequence reads were aligned to the *N. vitripennis* reference genome (Nvit_2.1, https://www.ncbi.nlm.nih.gov/assembly/GCF_000002325.3/) using the BWA-MEM algorithm (Version 0.7.15) (Li and Durbin 2009). The alignment files were converted to BAM format, sorted, and indexed using SAMtools (Version 0.1.19) (Li et al. 2009). FreeBayes (Version 1.9) (Garrison and Marth 2012) was used to detect genotyping variants. In total, 8,405,551 polymorphisms were identified, but the vast majority of mapped reads were spurious resulting from the inherently imperfect size selection step in creating the reduced representation library for GBS (Gileta et al. 2020). The result of this imperfect size selection is that off-target positions in the genome can be included, which we mitigated by strictly selecting on-target positions and filtering based on a high genotyping rate per SNP. Filtering was done using PLINK (Version 1.9) (Purcell et al. 2007) according to the following criteria: (i) read depth between 9 and 300, (ii) call rate greater than 80%, (iii) Hardy–Weinberg equilibrium (HWE) exact test *P*-value above 10^−4^, and (iv) minor allele frequency (MAF) higher than 0.02. Moreover, individuals with more than 30% missing SNPs were removed. After filtering, 8,639 SNPs remained across the whole genome of 1,230 individuals (717 in G169 and 513 in G172).

### Estimation of GEBVs with the GBLUP model

Genomic best linear unbiased prediction (GBLUP) was applied to predict GEBVs. GBLUP estimates the breeding values assuming that each marker explains an equal proportion of the total genetic variance (Meuwissen et al. 2001; VanRaden 2008). Thus, the statistical model used for GBLUP was as follows:


$$y=\mu +Xb+Z_{g}g+\left(\begin{array}{c} 0\\ Z_{c}c \end{array}\right)+\left(\begin{array}{c} e_{1}\\ e_{2} \end{array}\right),$$

where ***y*** is the vector of phenotypic records, *μ* is an intercept, ***b*** is a vector of fixed effects, ***X*** is a design matrix relating observations to the corresponding fixed effects, $Z_{g}$ is an incidence matrix that relates additive polygenic values (“breeding values”) to the animals, ***g*** is a vector of random additive polygenic effects of all individuals, **0** is a vector of 0s for the individuals in G169, ***c*** is a vector of random host effects for the individuals in G172, $Z_{c}$ is the corresponding incidence matrix, ***e***_1_ is a vector of random residuals for G169, and ***e***_2_ is a vector of random residuals for G172.

The additive and host effects were assumed to be normally distributed, as $g∼N(0,G\sigma_{g}^{2})$ and $c∼N(0,I\sigma_{c}^{2})$, respectively, where $\sigma_{g}^{2}$ and $\sigma_{c}^{2}$ are the additive genetic and host variances, and ***G*** is a matrix of additive genomic relationships between individuals. Allele frequencies of the current population were used to construct ***G***, following Method I of VanRaden (2008), $G=\frac{WW^{\prime }}{2\sum p_{j}(1-p_{j})}$, where $p_{j}$ is the allele frequency at locus *j*, and W is a matrix of centered allele counts, with elements $W_{ij}$ being the code of the genotype at locus *j* for individual *i*, with $\left(0-2p_{j}\right)$ for the homozygote, $\left(1-2p_{j}\right)$ for the heterozygote, and $\left(2-2p_{j}\right)$ for the opposite homozygote. Because the host effect could not be fitted for individuals from G169, variation originating from the host may end up in the residual variance for these individuals. For this reason, we estimated the residual variance for the G169 and G172 populations separately. The residual effects of G169 and G172 were assumed to be normally distributed, $e_{1}∼N(0,I\sigma_{e_{1}}^{2})$ and $e_{2}∼N(0,I\sigma_{e_{2}}^{2})$, respectively, where $\sigma_{e_{1}}^{2}$ and $\sigma_{e_{2}}^{2}$ are the residual variances of G169 and G172, respectively.

Because of the large host effect in *N. vitripennis*, especially for wing and body size traits, it is essential to include the host effect when estimating genetic parameters for size-related traits (Xia et al. 2020). Although we included data from both generations in the GBLUP, we estimated the genetic parameters for G172 only for which we could also estimate host effects. Heritabilities were calculated as follows:


$$h^{2}=\;\frac{\sigma_{g}^{2}}{\sigma_{p}^{2}},$$

where $\sigma_{p}^{2}=\sigma_{g}^{2}+\sigma_{c}^{2}+\sigma_{e_{2}}^{2}$ is the phenotypic variance of G172.

All analyses were performed using ASReml 4.0 (Gilmour et al. 2012), and the ***G*** matrix was calculated using Calc_grm (Calus and Vandenplas 2016).

### Population structure and LD estimation

The accuracy of GEBV predictions depends on the allele frequencies and the amount of recombination between loci in the sampled population. We therefore determined the presence of population structure across the sampled generations of the *N. vitripennis* HVRx population. We estimated the population structure among the 1,230 individuals of G169 and G172 by performing principal component analysis (PCA) based on the ***G*** matrix (Remington et al. 2001). Note that pedigree-based relationships between individuals were not available in this study. To visually inspect the presence of population structure, we visualized the genomic relationships between all individuals by plotting a heatmap of the ***G*** matrix, using superheat in R 4.4.1 (Barter and Yu 2018; R Core Team 2024). Analysis of the ***G*** matrix was done using ASRgenomics 1.1.3 in R (Gezan et al. 2021).

The amount of recombination between loci was determined by estimating the extent of LD between SNPs as $r^{2}$, using PLINK (Version 1.9) (Purcell et al. 2007). The decay of LD was estimated by fitting the equation $r^{2}=1/(1+px)$ for every focal SNP, using the nls method in R 4.4.1 (R Core Team 2024), where *x* denotes the distance to every other SNP.

### Validation

To assess the accuracy of genomic prediction, we performed cross-validation. In the cross-validation process, we divide the data set into 2 groups: a validation group where the phenotypes of the individuals are masked and a training group where both phenotypes and genotypes are used for genomic prediction. The training group was used to predict the GEBVs for individuals in the validation group, and the quality of genomic prediction is measured by the correlation between the GEBVs and the observed phenotypes in the validation group.

In this study, we performed 2 different ways of validation: across generation validation and 5-fold cross-validation using the entire population. In the across generation validation strategy, the phenotypes of 1 generation were masked and the GEBVs of these animals were predicted using the information from the other generation. Two different scenarios were applied here: (i) across generation forward validation (AGFV), using G169 to predict G172, and (2) across generation backward validation (AGBV), using G172 to predict G169. For the 5-fold cross-validation strategy, we randomly divided the population into 5 groups. In each cross-validation round, 1 group was taken as the validation group and the remaining 4 groups as the training population. The phenotypes in the validation group were masked, and the training group was used to predict GEBVs of individuals in the validation group. After 5 rounds, all individuals in all groups received predicted GEBVs. This procedure was repeated 50 times for each trait separately, and the results were averaged over 50 random divisions of the population into a validation group and a training group.

The expected value of the correlation between the GEBV and the phenotype of the validation individuals is equal to the product of heritability and the accuracy of the GEBV. Therefore, we estimated the accuracy of the GEBV by dividing the correlation between GEBVs and the observed phenotypes of individuals in the validation population by the square root of the heritability of the trait. The value of the accuracy can range from −1 to 1. While negative accuracies can be observed, the minimum accuracy is expected to be close to 0, occurring for example when a trait is not heritable or when the training population is small. High accuracies represent a strong correlation between the GEBV and the observed phenotype, indicating reliable genomic prediction, potentially increasing the rate of genetic improvement in genomic selection.

In addition to the accuracy of genomic prediction, we also evaluated the dispersion of the predicted GEBVs. The dispersion is measured by the regression coefficient of phenotypic observations on the predicted GEBVs in the validation population. A value of 1 is theoretically expected for correct estimates of GEBVs (correct dispersion). A deviation from 1 indicates an error in the scale of the GEBV: values greater than 1 indicate that the GEBV must be upscaled to achieve correct dispersion and therefore imply underdispersion of GEBVs. A regression coefficient smaller than 1 indicates that GEBVs must be downscaled and therefore implies overdispersion of GEBVs. While incorrect dispersion does not affect the accuracy of GEBVs and therefore also does not change the response to single-trait selection, it has practical relevance for genetic improvement. For example, overdispersion of GEBVs suggests a greater response to selection than can actually be achieved. Moreover, when selection is for multiple traits, a dispersion error will result in incorrect weighting of the traits in the multitrait selection index, which reduces the accuracy of the index and the response in the so-called aggregate genotype (for background on selection index theory, see e.g. Hazel et al. 1994).

## Results

### Population structure and LD

**Fig. 1.**
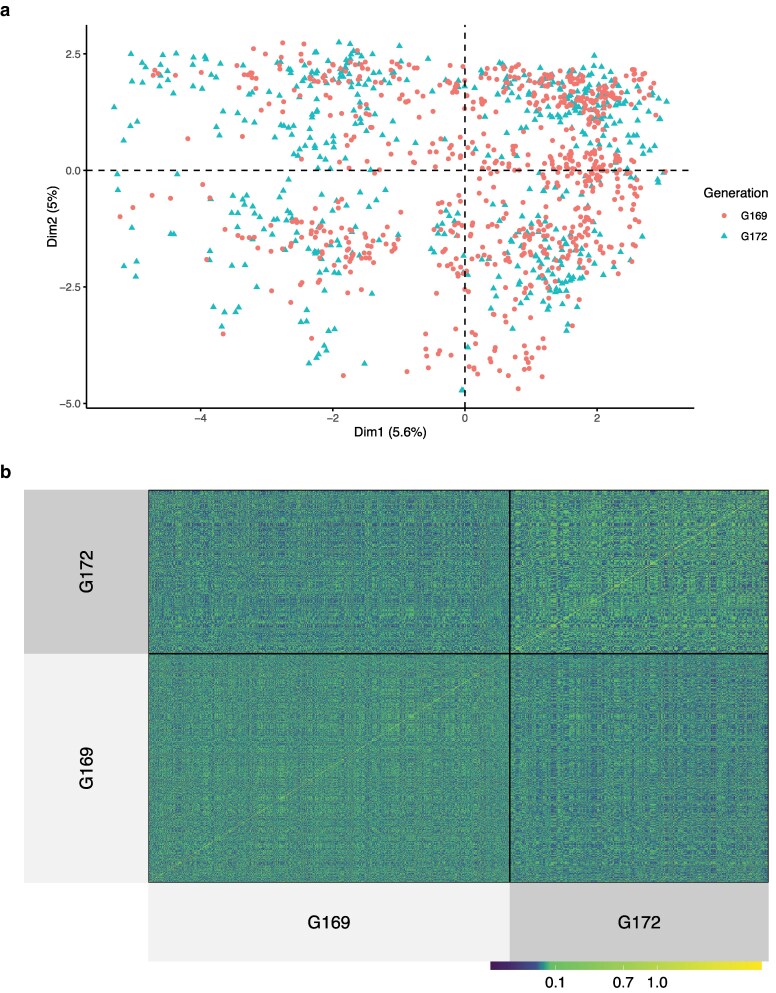
Genetic structure across G169 and G172 and relatedness among all individuals: a) PCA of G169 and G172, where the first 2 principal components explained 5.6% and 5.0% of variation, respectively. Dots represent individuals in G169, and triangles represent individuals in G172. b) The heatmap of the ***G*** matrix, in which high values indicate a close genomic relationship between 2 individuals, and values of 0 indicate no genomic relationship.

Population structure across G169 and G172 was assessed by PCA based on filtered SNPs. The PCA shows that the individuals from the 2 generations overlap almost completely, and the first 2 principal components explained only a small proportion of the total genetic variance, 6.13% and 5.27%, respectively (Fig. 1a). This suggests that individuals of G169 and G172 had very similar genotypes. The genomic relationships between all individuals were visualized using a heatmap of the ***G*** matrix (Fig. 1b). The heatmap suggests that most individuals were relatively unrelated, although some individuals had a higher relationship with each other. The average genomic relationship coefficient is close to 0: 0.000783 (standard deviation [SD] = 0.113) but has a wide range (Min: −0.474, Max: 1.49; Supplementary Fig. 1). This result is not unexpected because some individuals were collected from the same host and are therefore full or half-sibs with a high identity by descent. To investigate the LD ($r^{2}$) in our population, we plotted $r^{2}\;$ as a function of physical distance (Supplementary Fig. 2). We observed a slow decay of LD, with a half-decay distance of 34.1 kb and an average pairwise $r^{2}$ value of 0.47 (standard error [SE] = 0.0017) between loci separated by up to 1,000 kb.

### Validation of GEBVs

The heritability of all examined traits was moderate, ranging from $h^{2}=0.20$ for wing width to $h^{2}=0.27$ for second moment area (Table 2). For size-related traits, which are tibia length, wing length, wing width, and second moment area, the 5-fold cross-validation on the full data had a considerably higher accuracy than the across generation cross-validations, either forward or backward in time. For both AGFV and AGBV, the accuracies of genomic prediction were close to 0 for the size-related traits, indicating little predictive power. Remarkably, all estimated accuracies for size-related traits in AGFV were negative whereas positive in AGBV. The dimensionless wing aspect ratio showed a much higher accuracy for both across generation validations, 0.47 in AGFV and 0.65 in AGBV. The GEBV for all traits showed higher accuracies across all traits in the 5-fold cross-validation, with an average of 0.59 (SE = 0.03).

**Table 2.** Accuracy and dispersion^a^ of genomic prediction for tibia length and wing morphology traits in *N. vitripennis*, using 3 ways of validation with the GBLUP model: AGFV, AGBV, and 5-fold cross-validation.

Traits	$h^{2}$	AGFV		AGBV		5-fold cross-validation	
		Accuracy	Dispersion	Accuracy	Dispersion	Accuracy	Dispersion
Tibia length	0.21	−0.22	−0.21	0.21	2.21	0.52	0.96
Wing length	0.22	−0.12	−0.12	0.17	1.72	0.60	1.18
Wing width	0.20	−0.06	−0.06	0.22	1.62	0.67	1.19
Second moment area	0.27	−0.11	−0.11	0.06	0.34	0.61	1.07
Aspect ratio	0.22	0.47	0.73	0.65	1.48	0.55	0.79

Heritability $\left(h^{2}\right)$ is estimated for G172, based on the complete data set of both generations.

^a^A value of 1.0 of the regression coefficient represents the correct dispersion.

The regression coefficients of phenotypic observations on the GEBVs indicate possible dispersion in the prediction of the GEBVs. The regression coefficients of the across generation validations (forward and backward) deviated considerably from 1 (Table 2). However, because the accuracies of the GEBVs in both validations were close to 0, this does not provide meaningful information. In contrast, the regression coefficients for all traits were close to 1 in the 5-fold cross-validation (Table 2), which indicates correct estimation of GEBVs and little dispersion.

## Discussion

The need for genetic improvement of biocontrol agents has been pointed out by several authors. Despite its great success in livestock and plant breeding, genomic prediction has only been sparsely used in insects (Bernstein et al. 2023). Here, we performed genomic prediction for wing morphology traits using a GBLUP model in *N. vitripennis*, in a total of 1,230 individuals with GBS-based genotypes for 8,639 SNPs. Promising accuracies were observed for the GEBVs of all traits in the 5-fold cross-validation but not in both across generation validations (forward and backward). The regression coefficients in the 5-fold cross-validation indicated correct estimation of the GEBVs for all traits. Both accuracies and dispersion were in the range of those reported for honeybees (Bernstein et al. 2023).

The large difference between accuracies using different validation strategies and between traits seems to be related to the magnitude of the host effect. For size-related traits, host effects explained between 34% and 56% of the phenotypic variance, whereas for wing aspect ratio they explained only 8% (Xia et al. 2020). We did not observe a large host effect for wing aspect ratio because it was defined as the ratio of wing length and width, and thus the host effects on them had been scaled out. Moreover, the host effect in the estimation of the genetic parameters (see Materials and methods) appeared insufficient to uncover the standing genetic variation of the traits that are masked by strong variation in the host environment (Xia et al. 2020).

In AGFV, we had host information only for G172. Thus, host effects were absent in the training population. This likely resulted in the observed negative accuracies, which indeed suggests a flaw in the model.

In AGBV, the host information of G172 was included in the training population. It was therefore surprising to observe such low accuracies for size traits. Previous studies have reported that the accuracy of genomic prediction is determined by several factors, including the number of individuals in the training set population, the heritability of the trait, and the relatedness between the training and validation groups (Daetwyler et al. 2008). However, we did not observe a low accuracy for all traits in AGBV, with wing aspect ratio having an accuracy of 0.65. This suggests that the number of individuals in the training population and the relatedness between the training and validation groups are not causing observed low accuracies in the other traits. We therefore also checked the heritabilities for size traits in the G172 generation separately. When only G172 was included in the analysis, surprisingly low heritabilities for size traits were observed (Supplementary Table 1). These heritabilities are much lower than the values estimated from the combined data set, ranging from $h^{2}=0.20$ for wing width to $h^{2}=0.27$ for second moment area (Table 2). Furthermore, although we observed a lower heritability of wing aspect ratio in the separate G172 data set, it was not as much as for the size traits. Therefore, we conclude that the low heritability for size traits is likely the main cause for their low accuracies.

In contrast, in the 5-fold cross-validation we observed relatively high accuracies for all traits, ranging from 0.54 to 0.68. However, for the 5-fold validation, we randomly divided the individuals across the training and validation groups, which could lead to the training and validation groups including both full-sib individuals, causing inflation in prediction accuracy, a known problem in genomic prediction (Daetwyler et al. 2013). Because in the 5-fold cross-validation we included host effects for G172 in the prediction model and estimated heterogeneous residual variances for the 2 generations, we could (partially) account for the host effect. The accuracies in the 5-fold cross-validation are in the same range as those found in the 5-fold cross-validation for several traits in the honeybee (0.44 to 0.65 for beekeeper workability traits, such as gentleness, calmness, and swarming drive) (Bernstein et al. 2023). VanRaden et al. (2009) compiled the accuracy of genomic prediction for a number of traits in dairy cattle and found an average accuracy of about 0.7.

Compared to these genomic prediction studies in honeybees and dairy cattle, the accuracies in our study are very promising, given that we had a much smaller reference population size (less than 1,000 individuals compared to 2,970 in honeybees) and only moderate heritabilities (∼0.22). The most likely explanations for the relatively high accuracy are as follows: (i) the small genome size of *Nasonia*, with only 5 chromosomes and a genetic size of only 446.9 cM (Niehuis et al. 2010), and (ii) the high level of LD in our population. Together, both factors result in a limited effective number of independent chromosome segments ($M_{e}$) (Goddard 2009). Daetwyler et al. (2008, 2010) derived the relationship between $M_{e}\;$ and the accuracy of genomic prediction, $\rho_{g,\hat{g}}=\sqrt{N_{p}h^{2}/(N_{p}h^{2}+M_{e})}$, where $N_{p}$ is the number of individuals in the training population and $h^{2}\;$ is the heritability of the trait. We calculated $M_{e}\;$ following Goddard (2009), $M_{e}=1/Var(G_{i\neq j})$, where $Var(G_{i\neq j})$ denotes the variance among the off-diagonal elements of the genomic relationship matrix, resulting in $M_{e}=80$. Using this value in the above expression of expected accuracy resulted in even higher accuracies (∼0.85) than the values found in this study. This difference may occur because the markers may not fully capture all genetic variation and because the family structure in the population causes overprediction of accuracy based on $M_{e}$ (note that the Daetwyler equation is based on a population of “unrelated” individuals). Nevertheless, the low value of $M_{e}$ further supports our hypothesis that the high level of LD contributes to the relatively high accuracies found in this study.

In our population, LD decays relatively slowly ($r^{2}<0.1$ at 307.3 kb and $r^{2}<0.2$ at 136.5 kb). With *Nasonia* being a naturally inbreeding species, showing a high level of sib-mating (Luna and Hawkins 2004; Grillenberger et al. 2008; Buellesbach et al. 2023), slow decay of LD is expected. However, the estimates of LD decay in our population are almost double as previous estimates based on whole-genome sequencing of inbred lines from the same population (Pannebakker et al. 2020). The high level of LD or small $M_{e}$ in our population contributes to the promising accuracy in the 5-fold cross-validation because it has a relatively small effective population size ($N_{e}∼236$) (van de Zande et al. 2013). In applied biocontrol, the founder populations are commonly collected from the field, which may result in a large $N_{e}$ and thus a low level of LD and a high $M_{e}$. We have not found reports on population parameters, such as $N_{e}$ or $M_{e}$, for biocontrol agent populations so far. To maintain sufficient genetic variation, Bartlett (1993) recommended maintaining natural enemy populations with an $N_{e}>100$. If the $N_{e}$ of a population is indeed maintained close to 100, the application of genomic prediction in biocontrol agents can benefit from a good accuracy due to the relative low $N_{e}$ together with the small genome size.

### Challenges in biocontrol genomic selection

In addition to the accuracy, there are some other factors that need to be taken into consideration in practical applications of genomic prediction in biocontrol agents (and insects in general; Sandrock et al. 2025). First, many biocontrol agents are insects with a small body size, which may complicate DNA extraction and genotyping of single individuals and reduce the quality of the resulting genotype data. Moreover, when DNA extraction requires the entire individual, or a large proportion of its body, it is impossible to select the parents for the next generation from the genotyped individuals. Thus, currently, genomic selection seems more suitable for insect biocontrol agents with a bigger body size so that sufficient DNA can be obtained without sacrificing the entire individual. Second, the short generation interval of many insects may limit the benefits of genomic selection in biocontrol agents. One of the main advantages of genomic selection in animal breeding is the reduced generation interval, which increases genetic gain (Meuwissen et al. 2001). However, biocontrol agents such as *N. vitripennis* already have a short generation interval, and the use of genomic selection may not allow for an even shorter generation interval. Third, small biocontrol agents usually have a short lifespan. The time required for DNA isolation, genotyping, and breeding value estimation is often longer than the lifespan of the selection candidate. Thus, the application of genomic selection may be limited in organisms with a short lifespan. Yet, one could make clever use of the aspects of insect biology, such as the dependency of lifespan on temperature and the artificial induction of diapause, which both would enable buying time for genotyping and parent selection (cf. Pannebakker et al. 2008 and Zwaan et al. 1995). Finally, the current study has applied genomic prediction in a haplodiploid organism. While genomic prediction has originally been developed for diploid organisms (Meuwissen et al. 2001), the principle relies on estimating average effects of single marker alleles, which generalizes to haploid and polyploid individuals simply by adding the effects of all alleles at a locus (1 for haploids, >2 for polyploids). In the current study, we only used females so that our results are not impacted by the haplodiploid nature of the organism. Moreover, when the biocontrol trait of interest is expressed by females only, such as in the parasitization rate, while selection candidates are both males and females, then both the male and female parents for the next generation can simply be selected based on the estimated marker effects (or genomic breeding values) referring to the trait expressed by females. However, when both male and female phenotypes are relevant for biocontrol, such as in dispersal capacity, then it may be required to distinguish between marker effects (or genomic breeding values) expressed in males vs females. One approach is to use bivariate genomic prediction, where the male and female traits are treated as statistically distinct traits that are (genetically) correlated.

A more general issue in the genetic improvement of biocontrol agents, also challenging the application of genomic selection, is the difficulty in deciding which traits to optimize (Lommen et al. 2017; Bielza et al. 2020). Here, we focused on wing morphology as a relatively easy-to-measure model trait. Wing morphology has been shown to influence field dispersal and host location in parasitoids (Kölliker-Ott et al. 2003, 2004), with larger wings resulting in host parasitization over longer distances. As such, parasitoid dispersal capacity can be indicative to their efficacy as natural enemies, where the optimal dispersal rate depends on the agronomical context (Heimpel and Asplen 2011). Kruitwagen et al. (2018) listed several candidate biocontrol traits, including high killing efficiency, robustness under (a)biotic conditions in the area of release, environmental safety, and ability to be cost-effectively (mass) reared in the laboratory. However, efficient large-scale phenotyping methods for biocontrol traits still need to be developed. Moreover, studies on the genetic basis of these biocontrol traits (i.e. heritability) also are generally lacking (Lirakis and Magalhaes 2019, but see Chattington et al. 2025). Furthermore, many biocontrol agents are parasitoid wasps, which develop their offspring in a host with a set quality. Important biocontrol traits in parasitoids, such as sex ratio, female fecundity, and development time, are largely affected by the host quality (Godfray 1994). This further emphasizes the need to account for host quality in the application of these biocontrol agents.

Finally, the genotyping methods play an important role in the application of genomic selection in biocontrol agents. Here, we applied GBS as an economical method to obtain genome-wide marker genotypes from sequence data. A downside of GBS is the potential for relatively high rates of missing data (Poland and Rife 2012; Crossa et al. 2013), because it produces a reduced representation of the genome, which depends on the choice of restriction enzyme (de Ronne et al. 2023). We chose the *ApeKI* enzyme after in silico analysis (data not shown), and still our GBS protocol resulted in a lower marker density (8,639 SNPs) compared to whole-genome sequencing (205,691 SNPs) (Pannebakker et al. 2020). This resulted in larger gaps between markers, potentially missing recombination events, which most likely contributed to an overestimation of our LD measurements.

An alternative to GBS and whole-genome sequencing in insects would be the development of a SNP array, which provides a cost-effective, high-throughput option for screening large SNP numbers in large sample sizes. In insects, SNP arrays have already been developed for the dengue and yellow fever mosquito *Aedes aegypti* (50K SNPs) (Evans et al. 2015) and the honeybee *Apis mellifera* (>100K SNPs) (Jones et al. 2020). The initial costs for designing SNP arrays are high, but robust SNP arrays can be an economical alternative (down to €40 per sample) (Suratannon et al. 2020) for large sample sizes. A promising alternative to SNP arrays in insects is using low-coverage whole-genome sequence data, in combination with imputation to call genotypes. When used on the Oxford Nanopore MinION platform at coverages as low as 0.1×, this method can bring the cost of genotyping down to $40 per sample and provide results within 24 h (Lamb et al. 2023).

The abovementioned factors make genomic selection for biocontrol agents currently a difficult and technological application. However, with the fast development of techniques, such as larger-scale phenotyping and genotyping methods and improved computer algorithms, the present challenges will likely be overcome in the near future. Nevertheless, our results provide a realistic assessment of the potential benefits of genomic selection when applied to natural enemies to improve biocontrol efficacy, as well as for future research. Although this study mainly focuses on application in the parasitoid *N. vitripennis*, our findings and discussion of the limitations also apply to the potential application of genomic selection in insects for other uses.

## Supplementary Material

jkaf285_Supplementary_Data

## Data Availability

Insect material is available upon request. The authors affirm that all data necessary for confirming the conclusions of the article are present within the article, figures, and tables. Sequence reads are deposited in the NCBI Short Read Archive under BioProject accession number PRJNA1188512. SNP data in VCF format and wing and tibia measurements are deposited at DANS Data Station Life Sciences under doi: 10.17026/LS/GE5QSB. Supplemental material available at G3 online.
