# Supplementary material for: Genomic prediction in insects: a case study on wing morphology traits in the jewel wasp Nasonia vitripennis
Source: G3 (Bethesda). 2025 Nov 29;16(2):jkaf285. doi: 10.1093/g3journal/jkaf285 (PMC12869063; doi:10.1093/g3journal/jkaf285)
Supplement: jkaf285_Supplementary_Data [file jkaf285_supplementary_data.zip › Supplemental_Figures_G3-2025-406234.pdf]

Supplementary Figures

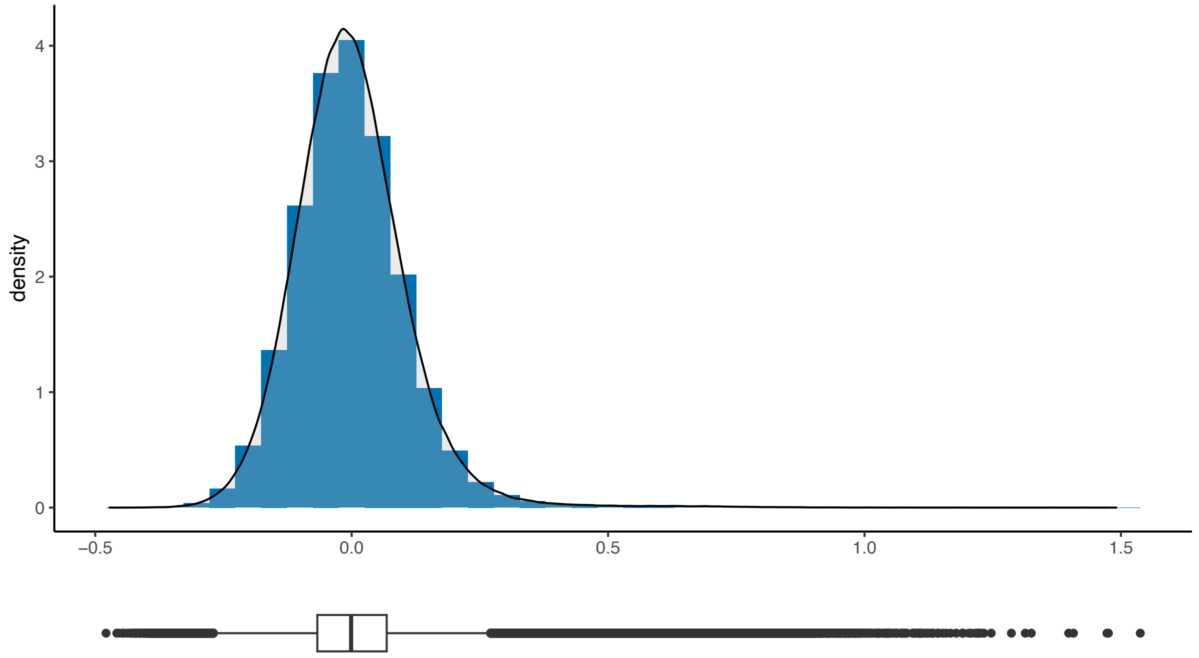

Supplementary Figure 1. Density plot of the distribution off-diagonal values of the ***G*** matrix, indicating the genomic relationships between individuals across generations G169 and G179.

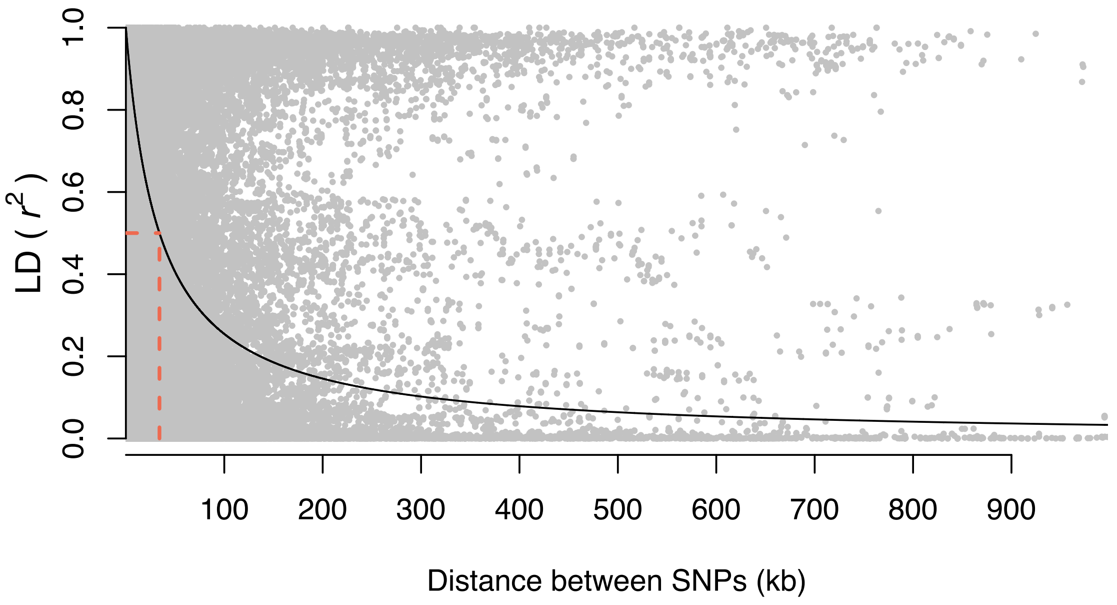

Supplementary Figure 2. Decay of linkage disequilbrium (LD) with physical distance. Points represent the r^2 for pairs of markers, solid black line gives the non-linear least squares fit of r^2 on the distance between pairs of SNP. Dashed line indicates the half-decay LD distance at 34.1 kb.
