# Supplementary material for: Genomic prediction in insects: a case study on wing morphology traits in the jewel wasp Nasonia vitripennis
Source: G3 (Bethesda). 2025 Nov 29;16(2):jkaf285. doi: 10.1093/g3journal/jkaf285 (PMC12869063; doi:10.1093/g3journal/jkaf285)
Supplement: jkaf285_Supplementary_Data [file jkaf285_supplementary_data.zip › Supplemental_Tables_G3-2025-406234.docx]

**Supplementary Table S1.** Estimated variance components for wing morphology traits and tibia length, based on G172 only.

| Traits | $\sigma_{g}^{2}$ | $\sigma_{c}^{2}$ | $\sigma_{e}^{2}$ | $\sigma_{p}^{2}$ | $h^{2}$ | $c^{2}$ |
| --- | --- | --- | --- | --- | --- | --- |
| tibia length (µm) | 33.15 | 644.87 | 298.29 | 976.31 | 0.03 (0.03) | 0.66 (0.04) |
| wing length (µm) | 226.12 | 6334.62 | 1987.41 | 8548.2 | 0.03 (0.03) | 0.74 (0.03) |
| wing width (µm) | 84.44 | 1597.19 | 432.22 | 2113.8 | 0.04 (0.03) | 0.76 (0.03) |
| second moment area (mm4) | 4.60×10-4 | 5.42×10-3 | 1.91×10-3 | 7.79×10-3 | 0.06 (0.03) | 0.70 (0.04) |
| aspect ratio (-) | 1.64×10-4 | 1.58×10-4 | 9.17×10-4 | 1.24×10-3 | 0.13 (0.06) | 0.13 (0.05) |

$\sigma_{g}^{2}$: additive genetic variance; $\sigma_{c}^{2}$: variance of host effects; $\sigma_{e}^{2}$: residual variance of G172; $\sigma_{p}^{2}$: phenotypic variance of G172, calculated as $\sigma_{p}^{2}=\sigma_{g}^{2}+\sigma_{c}^{2}+\sigma_{e}^{2}$; $h^{2}$: heritability of G172, calculated as $h^{2}=\frac{\sigma_{g}^{2}}{\sigma_{p}^{2}}$, standard deviation in parentheses; $c^{2}$: proportion of variance due to host effects of G172, calculated as $c^{2}=\frac{\sigma_{c}^{2}}{\sigma_{p}^{2}}$, standard deviation in parentheses.

Variance components were estimated using data from G172 only, and the following statistical model:

$\boldsymbol{y}=\mu+\boldsymbol{Xb}+\boldsymbol{Z}_{g}\boldsymbol{g}+\boldsymbol{Z}_{\boldsymbol{c}}\boldsymbol{c}+e$,

where ***y*** is the vector of phenotypic records for G172, $\mu$ is an intercept, b is a vector of fixed effects, ***X*** is a design matrix relating observations to the corresponding fixed effects. $\boldsymbol{Z}_{\boldsymbol{g}}$ is an incidence matrix that relates additive polygenic values (“breeding values”) to the animals, ***g*** is a vector of random additive polygenic effects of all individuals, ***c*** is a vector of random host effects for the individuals in generation G172, $\boldsymbol{Z}_{\boldsymbol{c}}$ is the corresponding incidence matrix, ***e*** is a vector of random residuals for G172.
